# Cardiac arrhythmia spot light: Detection of ventricular fibrillation—Not all ICDs are created equal

**DOI:** 10.1002/joa3.12554

**Published:** 2021-05-19

**Authors:** Adam Lee, James W. Salazar, Zian H. Tseng

**Affiliations:** ^1^ Department of Medicine Section of Cardiac Electrophysiology Division of Cardiology University of California San Francisco CA USA; ^2^ Department of Medicine University of California San Francisco CA USA

## Abstract

Intracardiac device electrograms demonstrating differing VF sensing performance between two manufacturer ICD generators.

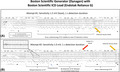

## 1 CASE DESCRIPTION

A 52‐year‐old woman presented for generator replacement of a Boston Scientific dual‐chamber implantable cardioverter‐defibrillator (ICD). It had been placed 10 years earlier for secondary prevention of idiopathic ventricular fibrillation (VF), with last appropriate shock 3 years prior. Although ventricular sensing was adequate during routine in‐clinic device interrogation, when measured intra‐operatively, the R‐waves were of borderline voltage (<5 mV) raising concerns about the device's ability to sense VF.

**FIGURE 1:**
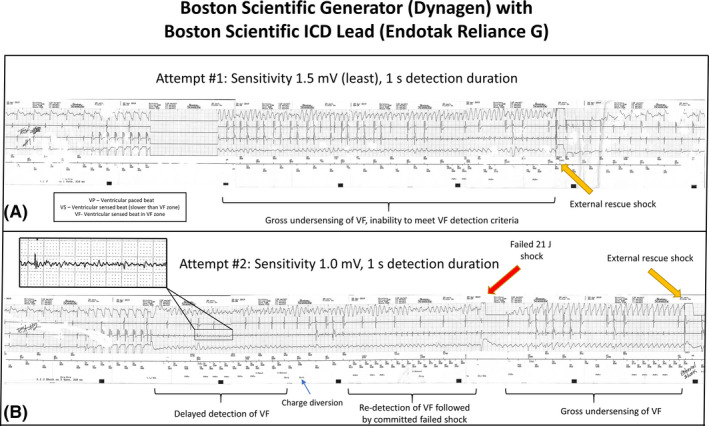
Intracardiac electrograms during defibrillation threshold testing (Boston Scientific Generator)

**FIGURE 2:**
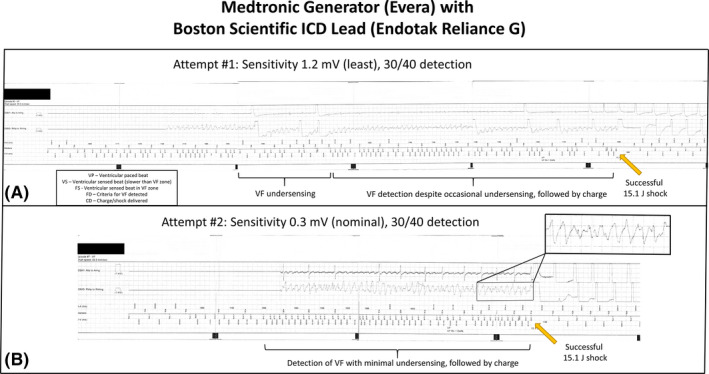
Intracardiac electrograms during defibrillation threshold testing (Medtronic Generator)

After replacement with a new generator (Boston Scientific Dynagen), defibrillation threshold (DFT) testing was performed to confirm adequate VF sensing. VF was induced and VF sensing assessed at least sensitivity (1.5 mV). VF was completely undersensed by the ICD as indicated by the device attempting to pace the ventricle during VF (Figure 1A). An external rescue shock was required. VF induction was repeated, and the ICD programmed to a higher sensitivity (1.0 mV), but significant VF undersensing remained resulting in charge diversion occurring following initial VF detection. When VF was re‐detected, a 21 J shock failed to convert the patient to sinus rhythm and an external shock was again required (Figure 1B) as VF post‐shock remained undetected because of gross undersensing. Thus, an adequate safety margin for VF detection was not established. A generator from an alternative manufacturer (Medtronic Evera XT) was connected to the existing Boston Scientific leads. DFT testing was repeated at least sensitivity (1.2 mV for this device) which resulted in some degree of under‐sensing, though ultimately successful VF detection and defibrillation (Figure 2A). Repeat testing at nominal sensitivity (0.3 mV) demonstrated satisfactory VF detection (Figure 2B).

The patient underwent replacement with the Medtronic generator with shortened detection intervals (18/24 intervals) to compensate for potential VF undersensing. The Boston Scientific generator was returned to the manufacturer for analysis. The final report determined that the “…ICD was operating normally as programmed and designed.”

## 2 DISCUSSION

Major society guidelines recommend against DFT testing in routine left‐sided transvenous ICD implantations, in view of recent large multi‐center randomized controlled trials (SIMPLE, NORDIC) that demonstrated no clinical outcome benefits of DFT testing and a small but definitive risk of adverse events arising from VF induction, anesthesia, and shocks. Thus, unless DFTs are expected to be elevated (eg right‐sided transvenous ICDs, hypertrophic cardiomyopathy), DFT testing is commonly omitted at the time of ICD procedures. However, DFT testing, in spite of the nomenclature implication, serves a second important purpose—ensuring adequate sensing of VF. Failure to sense VF was the most common cause of death in patients with ICDs in our systematic postmortem examination of all such sudden deaths over 3 years in the countywide POST SCD Study.^1^ Although the relationship between intrinsic sinus R‐wave and VF sensing is imperfect, current guidelines recommend achieving a sensed R‐wave amplitude of >5 to 7 mV to ensure adequate VF sensing.

Direct comparison of sensing characteristics and behavior between different manufacturer ICD generators is rare. At a similar sensitivity (1.5 vs 1.2 mV), the Boston Scientific generator completely under‐sensed VF with short detection criteria while the Medtronic generator successfully detected VF using the same lead with minimal under‐sensing and no charge diversion despite prolonged detection intervals (Table 1). These observations, suggest that manufacturer‐specific algorithms, including differing filter and gain control settings, translate to meaningful differences in reliable VF detection.

**TABLE 1:** Hardware, programming, and outcomes for the four defibrillation threshold test events

ICD Generator	Attempt no.	Sensitivity (mV)	Detection	VF Detection
Boston Scientific Dynagen	1	1.5 (least)	1 second (nominal)	Failed
	2	1.0	1 second (nominal)	Successful on redetection^a^
Medtronic Evera	1	1.2 (least)	30/40 (prolonged)	Successful
	2	0.3 (nominal)	30/40 (prolonged)	Successful

^a^ Charge diversion occurred on initial detection because of gross VF undersensing that resulted in a significant delay to therapy delivery.

Two factors may have contributed to the failure of the Boston Scientific generator to sense VF: (a) the lower nominal RV lead sensitivity and (b) the specific automatic gain control (AGC) algorithm.

ICDs rely on auto‐adjusting sensitivities that initially set a high detection threshold (to avoid oversensing pacing stimuli, the evoked potential, the T‐wave, etc), which subsequently decays to a lower detection threshold to allow sensing of low amplitude VF. Both companies’ algorithms set the initial post‐blanking sensitivity at 75% of the preceding sensed peak value. However, Medtronic's algorithm caps the maximum threshold at 8x the programmed sensitivity value (or 4.5x after a paced beat), while the Boston Scientific AGC algorithm does not. This difference may render the AGC algorithm more prone to under‐sensing polymorphic rhythms which can have wide variability in electrogram amplitude. Boston Scientific RV/ICD leads rely solely on integrated bipolar sensing; these features may be useful in filtering unwanted far‐field signal (eg, diaphragmatic myopotentials) but at the cost of reliable sensing of low amplitude signals during VF. Our experience mirrors other reports of VF under‐sensing by ICDs utilizing AGC despite “adequate” sensed R‐waves in sinus rhythm, including our postmortem series.^1^, ^2^, ^3^ Conversely, a study examining Medtronic ICDs with standard auto sensitivity adjustment demonstrated excellent VF detection even with sensed R‐waves during sinus rhythm as low as 3 mV,^4^ though these systems also utilized dedicated bipolar sensing.

Although multiple randomized controlled trials have demonstrated morbidity and mortality benefit of prolonged ICD detection to avoid inappropriate and premature ICD therapies, such programming is not appropriate in all circumstances. In patients with unreliable sensing of ventricular arrhythmias, it is clearly detrimental. Indeed, nearly all of the fatal under‐sensing events in our postmortem experience were observed in ICDs programmed in this manner.^1^ Prolonged detection intervals compound delays to detection because of VF under‐sensing, leading to a vicious cycle of deteriorating electrogram amplitudes in VF and lower chance of defibrillation success because of prolonged hypoxia and myocardial ischemia, particularly beyond the controlled setting of DFT testing.

## 3 CONCLUSIONS

Although DFT testing can likely be safely omitted in routine ICD generator changes, inadequate R‐wave sensing in sinus rhythm (<5 to 7 mV), even if intermittent, should prompt DFT testing to assess the adequacy of VF sensing. In the event of inadequate sensing during VF, switching to an alternative manufacturer's generator before proceeding to lead revision may be a reasonable bridging solution if an adequate safety margin can be demonstrated. Prolonged interval detection should not be programmed in patients with unreliable VF sensing. Improving VF sensing and detection represents an opportunity for manufacturer design, algorithm improvement, and clinical practice improvement, with the goal of further optimizing ICD performance to rescue patients from sudden death.

## CONFLICT OF INTEREST

Dr Lee has no disclosures of relevance. Drs Salazar (R38HL143581) and Tseng (R01HL102090, R01HL126555, R01HL147035) reported grants from the National Heart Lung and Blood Institute, and Centers for Disease Control and Prevention (1NU38DP000019‐01‐00).

## References

[1] Tseng ZH , Hayward RM , Clark NM , Mulvanny CG , Colburn BJ , Ursell PC , et al. Sudden death in patients with cardiac implantable electronic devices. JAMA Intern Med. 2015;175(8):1342–50. 26098676 10.1001/jamainternmed.2015.2641

[2] Chin A , Healey JS , Ribas CS , Nair GM . Delayed AICD therapy and cardiac arrest resulting from undersensing of ventricular fibrillation in a subject with hypertrophic cardiomyopathy–a case report. Indian Pacing Electrophysiol J. 2015;15(2):121–4. 26937098 10.1016/j.ipej.2015.07.009 PMC4750165

[3] Dekker LR , Schrama TA , Steinmetz FH , Tukkie R . Undersensing of VF in a patient with optimal R wave sensing during sinus rhythm. Pacing Clin Electrophysiol. 2004;27(6 Pt 1):833–4. 15189548 10.1111/j.1540-8159.2004.00542.x

[4] Ruetz LL , Koehler JL , Brown ML , Jackson TE , Belk P , Swerdlow CD . Sinus rhythm R‐wave amplitude as a predictor of ventricular fibrillation undersensing in patients with implantable cardioverter‐defibrillator. Heart Rhythm. 2015;12(12):2411–8. 26272520 10.1016/j.hrthm.2015.08.012

